# 

**Published:** 1874-05

**Authors:** 


### NOTICES FOR MAY.

SOME have written to the Editor of the non-reception of the JOURNAL. In all such cases it has been found on inquiry, that the letters were not sent either to the Editor's office or that of the Publishers, E. H. Gibbs & Co., 84 Broadway, New York, to whom all letters in reference to the JOURNAL should be sent. But as the size and price of the JOURNAL have been doubled, and such parties have sent but one dollar, if they will send another dollar all the numbers for 1874 will be sent; otherwise, by sending their names, all the numbers to June, inclusive, will be sent for the dollar already forwarded. All letters from the Editor, professional or otherwise, are signed W. W. Hall.

SEWING MACHINES.—It is well ascertained that temporary, and even permanent injury has resulted in many cases from the steady use of the treadle several hours at a time every day.

The SEWING MACHINE ENGINE COMPANY, 841 Broadway, New York, have patented a "Motor" which, without any danger, and at an expense of two cents an hour for gas or oil, will run a machine the whole day, thus enabling the operator to do a larger amount of work with much less fatigue, and avoiding all risk whatever from the use of the treadle. The price of this "Motor" is forty dollars.

THE Second Annual Report of the ROOSEVELT HOSPITAL has been presented by Horatio Paine, M. D., Superintendent, showing that over one thousand males and females have been treated for various ailments during 1873, of whom one half were cured, and only twelve per cent. died. The generous founder of this magnificent charity had all the prospects of life blighted by having been made a cripple, and, no doubt, in the solitude of his own chamber he was led to cherish sympathies for the suffering poor, which led him to leave almost his entire fortune for the alleviation of those who might come after, and who will have reason to revere his name and memory down through the ages. The splendid building is completed, and its doors are open to persons of every clime, and caste, and country.

Any person giving three thousand dollars can nominate a patient to a bed without charge during the donor's life time. Five thousand dollars extends that privilege to the donor's heirs and assigns in perpetuity. M. Trimble, 59 East 25th street, Treasurer. The hospital is within a block or two of the 8th Avenue entrance to Central Park, and within view of the beautiful Hudson River.

"SKIN GRAFTING."—R. J. Levis, M. D., Surgeon to the Pennsylvania Hospital and to the Wills Ophthalmic Hospital of Philadelphia, has published a pamphlet on the method of placing bits of healthy skin over spaces where the natural skin has been removed, which take root and grow and spread. There is reason to believe that, as additional skill is acquired, persons more or less bald may be made to have a good "head of hair."

"PHRENOLOGICAL JOURNAL," $3 a year; 389 Broadway, New York. The increasing practical and scientific value of this well known monthly commends it to the extended patronage of an intelligent public.

☞ "THE HERALD OF HEALTH," devoted to the culture of body and mind, $2 a year, No. 15 Laight street, New York, is steadily improving in the character of its articles; every number contains a great variety of practical thoughts in reference to the conduct of life.

Extra No. 15 of the New York *Tribune* contains more valuable reading for 20 cents than was ever given before in pamphlet form in our memory. Discoveries on the site of Ancient Troy, by Bayard Taylor; Brown-Sequard's Lectures on the Nerves, Sumner's Sufferings, Proctor's Astronomical Lectures, and the Germ Theory of Disease, by Professor C. F. Chandler.

